# Supplementary material for: Bioinformatics-Based Identification of Expanded Repeats: A Non-reference Intronic Pentamer Expansion in RFC1 Causes CANVAS
Source: Am J Hum Genet. 2019 Jun 20;105(1):151–65. doi: 10.1016/j.ajhg.2019.05.016 (PMC6612533; doi:10.1016/j.ajhg.2019.05.016)
Supplement: Document S1. Figures S1–S7 and Tables S1–S4 [file mmc1.pdf]

The American Journal of Human Genetics, Volume *105*

Supplemental Data

Bioinformatics-Based Identification of Expanded Repeats: A Non-reference Intronic Pentamer Expansion in *RFC1* Causes CANVAS

Haloom Rafehi, David J. Szmulewicz, Mark F. Bennett, Nara L.M. Sobreira, Kate Pope, Katherine R. Smith, Greta Gillies, Peter Diakumis, Egor Dolzhenko, Michael A. Eberle, María García Barcina, David P. Breen, Andrew M. Chancellor, Phillip D. Cremer, Martin B. Delatycki, Brent L. Fogel, Anna Hackett, G. Michael Halmagyi, Solange Kapetanovic, Anthony Lang, Stuart Mossman, Weiyi Mu, Peter Patrikios, Susan L. Perlman, Ian Rosemergy, Elsdon Storey, Shaun R.D. Watson, Michael A. Wilson, David S. Zee, David Valle, David J. Amor, Melanie Bahlo, and Paul J. Lockhart

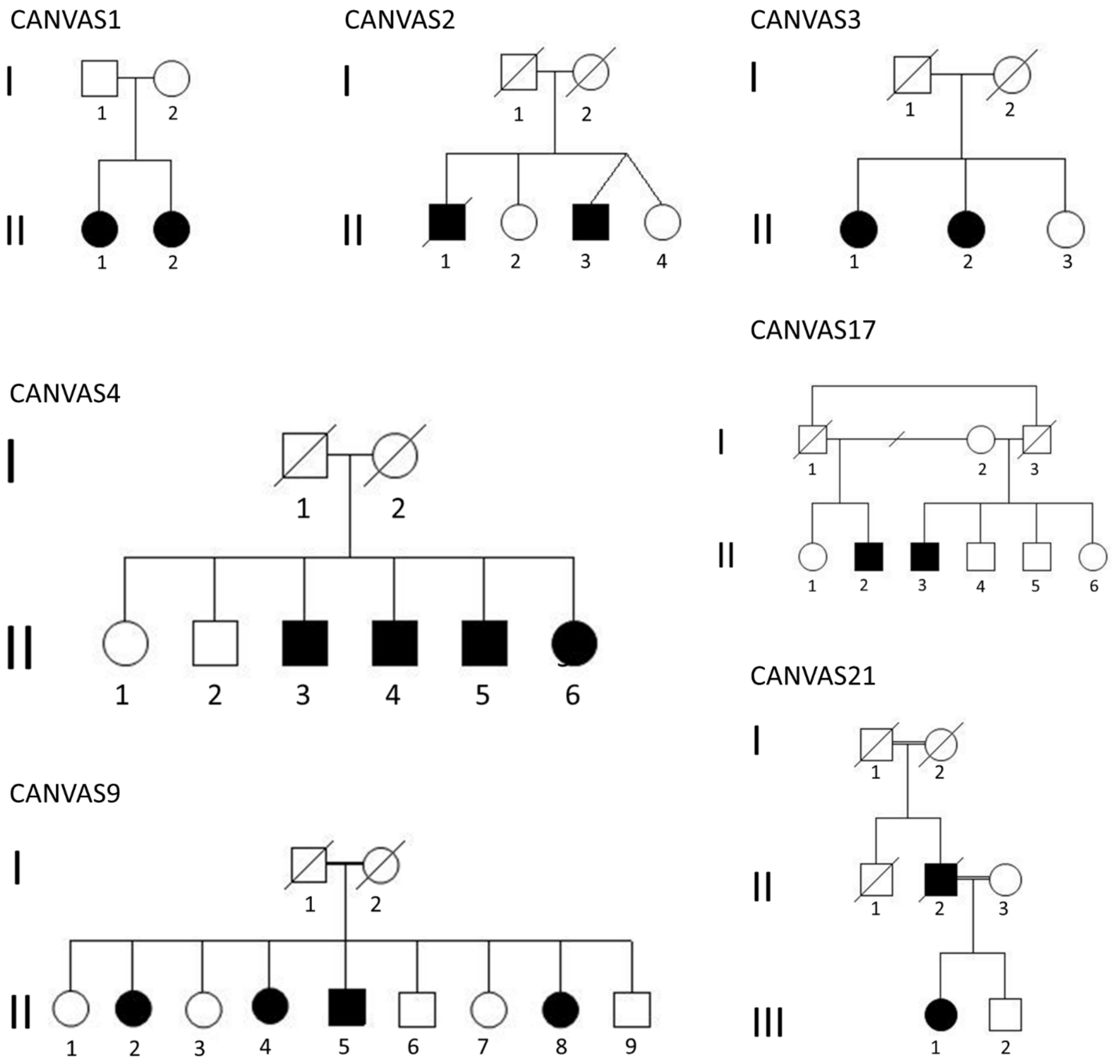

Figure S1: Pedigree structure of CANVAS families utilized for linkage studies (CANVAS1, 2, 3, 4 and 9) or demonstrating multigenerational inheritance.

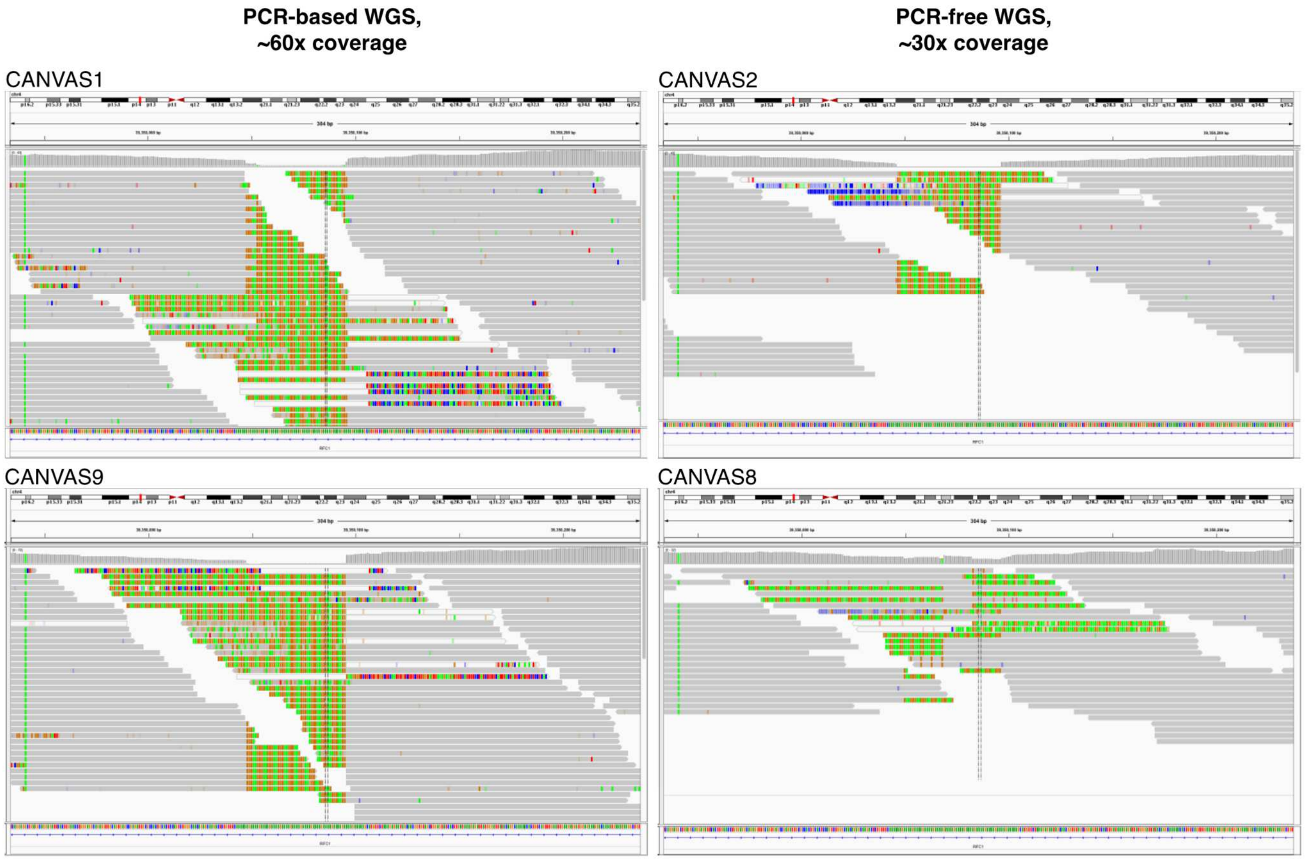

Figure S2: IGV snapshots of the (AAGGG)n locus in *RFC1*.

Illustrated are affected individuals from homozygous carriers of AAGGG (CANVAS1, 2, 9) and an individual who carries the AAGGG allele in addition to an expanded AAAGG allele (CANVAS8).

A

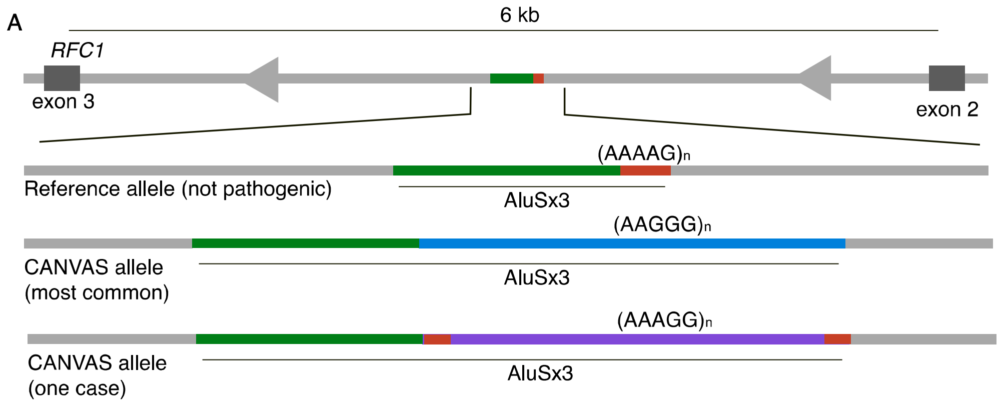

B

Individual ID	Allele	Pathogenic Motif	Sequence
REFERENCE			GACTCTGTTTCAAAAAAGAAAAGAAAAGAAAAGAAAAGAAAAGAAAAGAAAAGAAAAG- - AAAAGAAAAGAAAAGAAAAGAAAAGAAAAGAAAAGAAAAAGCAT
CANVAS9-10	A1/2	AAGGG	GACTCTGTTTCAAAAAGGGAAGGGAAGGGAAGGGAAGGGAAGGGAAGGGAAGGG- - - AAGGGAAGGGAAGGGAAGGGAAGGGAAGGGAAAGAAAAAGCAT
CANVAS8-8	A1	AAGGG	GACTCTGTTTCAAAAAAGGAAGGGAAGGGAAGGGAAGGGAAGGGAAGGGAAGGG- - - AAGGGAAGGGAAGGGAAGGGAAGGGAAGGGAAGAAAAAGCAT
CANVAS8-8	A2	AAAGG	GACTCTGTTTCAAAAAAGAAAAGAAAAGAAAAGAAAAGAAAAGAAAAGAAAAGAAAGG- - AAAGGAAAAGAAAAGAAAAGAAAAGAAAAGAAAAGAAAAAGCAT
CANVAS2-2	A1	AAGGG	GACTCTGTTTCAAAAGGAAAGGGAAGGGAAGGGAAGGGAAGGGAAGGGAAGGG- - - - AAGGGAAGGGAAGGGAAGGGAAGGGAAGGGAAGAAAAAGCAT
CANVAS2-2	A2	AAGGG	GACTCTGTTTCAAAAGGAAAGGAAAGGGAAGGGAAGGGAAGGGAAGGGAAGGG- - - - AAGGGAAGGGAAGGGAAGGGAAGGGAAGGGAAGAAAAAGCAT
CANVAS1-1	A1	AAGGG	GACTCTGTTTCAAAAAAGAAAGGGAAGGGAAGGGAAGGGAAGGGAAGGGAAGGG- - - AAGGGAAGGGAAGGGAAGGGAAGGGAAGGGAAGAAAAAGCAT
CANVAS1-1	A2	AAGGG	GACTCTGTTTCAAAAAGGGAAGGGAAGGGAAGGGAAGGGAAGGGAAGGGAAGGG- - - AAGGGAAGGGAAGGGAAGGGAAGGGAAGGAAAGAAAAAGCAT

Figure S3: Schematic representation of pathogenic CANVAS alleles.

(A) Visual representation of the AAGGG and AAAGG repeat expansions in the second intron of *RFC1*. (B) Visual representation of the different AAGGG/AAAGG alleles in affected individuals identified by WGS.

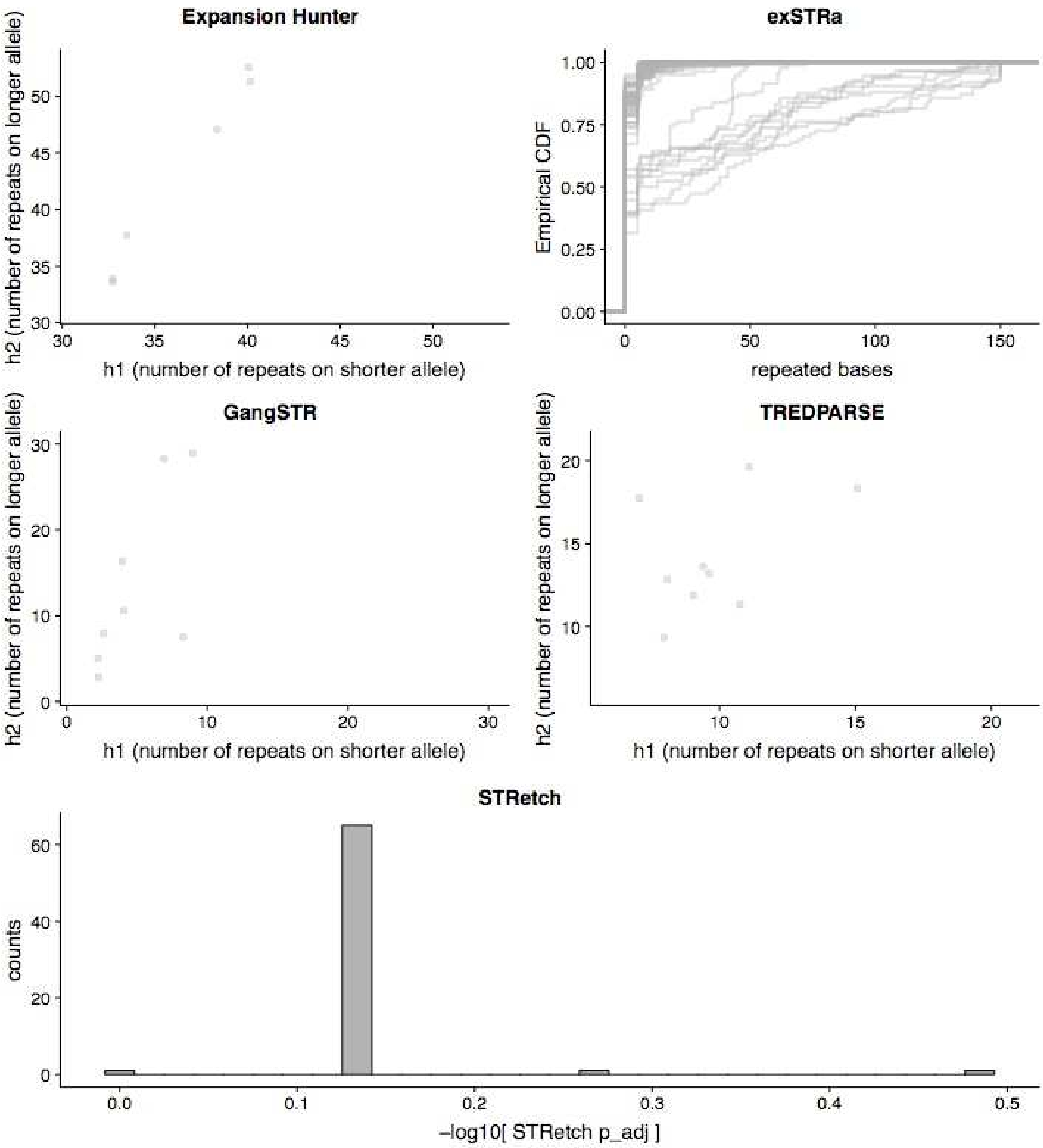

Figure S4: Computational validation of the $(AAGGG)_n$ STR.

The WGS from 69 unrelated non-CANVAS individuals (Coriell dataset) were analysed at the coordinate's chr4:39350045-39350095, using the tools exSTRa, EH, GangSTR, TREDPARSE and STRetch.

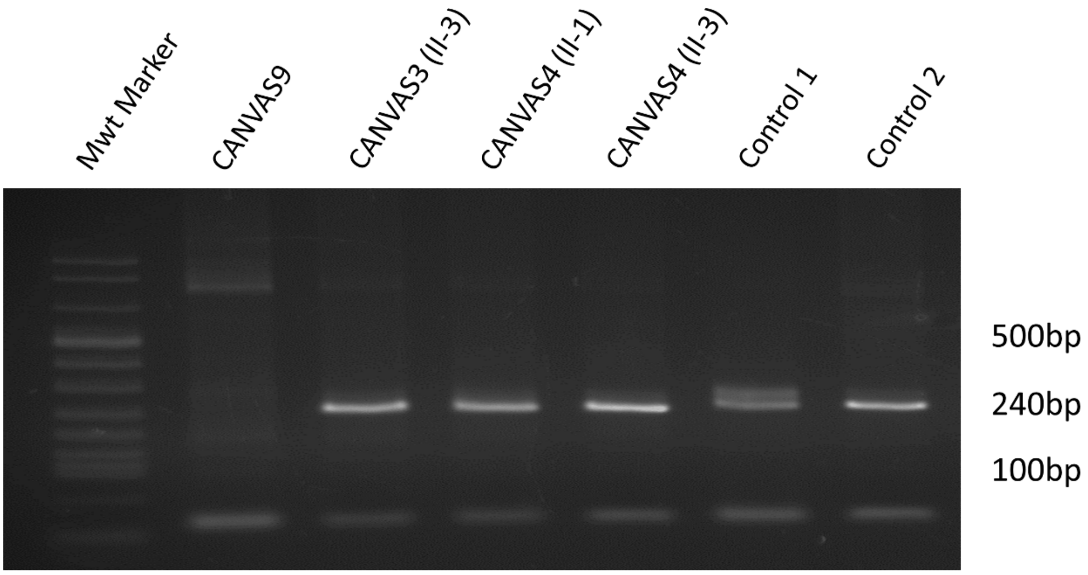

Figure S5: PCR analysis of unaffected individuals from *RFC1* linked CANVAS families. Genomic DNA from three unaffected family members from CANVAS3 and CANVAS4 was analysed for the presence of a non-expanded *RFC1* allele. All three individuals carried at least one non-expanded allele, as indicated by the ~250bp PCR product. CANVAS9 is homozygous for the expanded allele and indicates the pattern expected in the absence of the reference allele. Pedigree structure and affected status are illustrated in Figure S1.

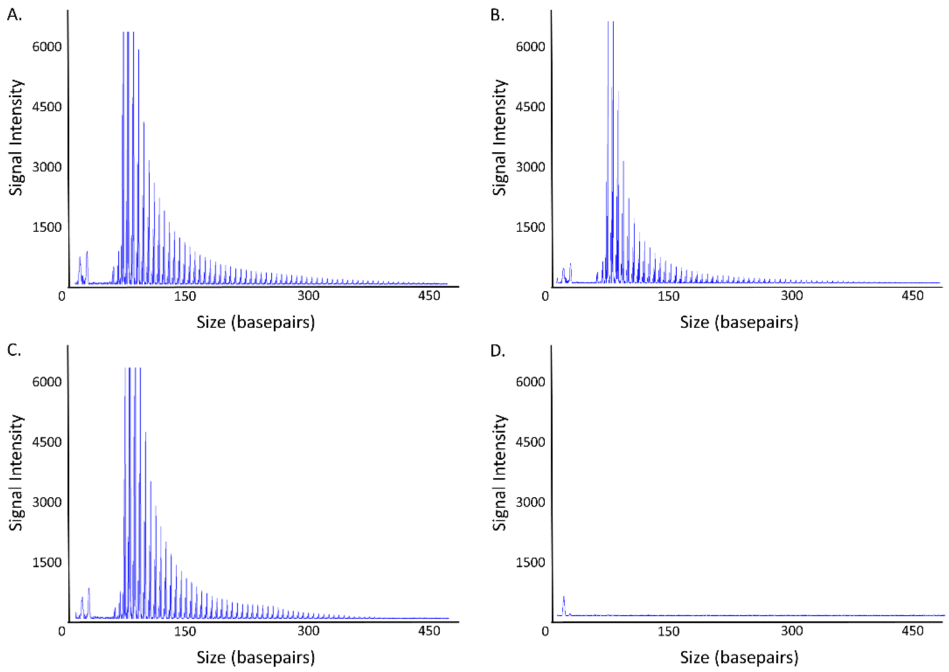

Figure S6: Repeat-primed PCR analysis of unaffected individuals with (AAGGG)exp RE. Representative images of the repeat-primed PCR for the (AAGGG)exp RE demonstrating a saw-toothed product with 5 base pair repeat unit size, amplified from gDNA of control individuals with heterozygous (A, B) or homozygous (C) alleles encoding the (AAGGG)exp RE. No product was observed for the no gDNA template negative control (D).

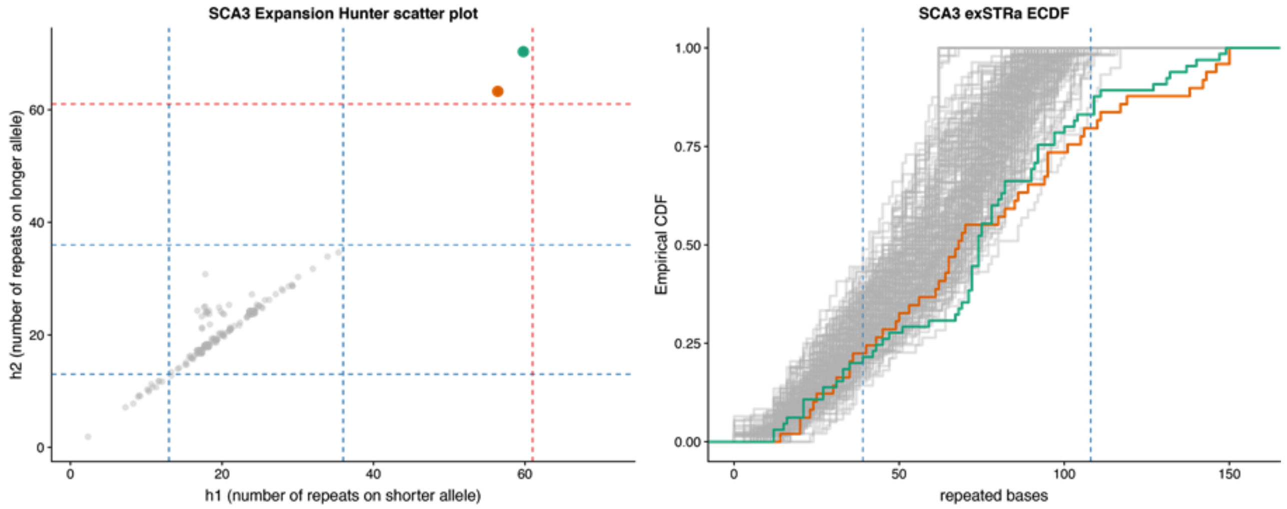

Figure S7: Identification of a pathogenic SCA3 expansion in CANVAS13.

The WGS data for SCA3 (green dot) and an individual with a confirmed SCA3 RE (orange dot) was analysed using ExpansionHunter and exSTRa. This analysis identified a heterozygous, pathogenic SCA3 expansion in CANVAS13.

Primer name	Position (hg19)	Sequence
CANVAS_RFC1_3F	chr4: 39350172-39350192	5'-ACTGACAGTGTTTTTGCCTGT
CANVAS_RFC1_3R	chr4:39349940-39349959	5'-GGCTGAGGCAGGAGATTCAC
TPP_CANVAS_FAM_2F	chr4:39350172-39350192	FAM-5'-ACTGACAGTGTTTTTGCCTGT
5R_TPP_M13R_CANVAS_RE_R	NA	5'-CAGGAAACAGCTATGACC_AAGGGAAGGGAAGGGAAGGGAAGGG
TPP_M13R	NA	5'-CAGGAAACAGCTATGACC

Table S1: Primer sequences for analysis of RFC1.

The gene reference sequences utilized were NC_000004.11 and NM_002913.4 (*RFC1*).

ENSEMBL Gene ID	Gene name	Gene biotype	OMIM gene ID	OMIM disease phenotype (phenotype, OMIM phenotype ID, inheritance pattern)
ENSG00000121895	TMEM156	protein_coding	NA	NA
ENSG00000109790	KLHL5	protein_coding	608064	NA
ENSG00000249207	RP11-360F5.1	antisense	NA	NA
ENSG00000249685	RP11-360F5.3	lincRNA	NA	NA
ENSG00000157796	WDR19	protein_coding	608151	?Cranioectodermal dysplasia 4, 614378, AR; ?Short-rib thoracic dysplasia 5 with or without polydactyly, 614376, AR;Nephronophthisis 13, 614377, AR; Senior-Loken syndrome 8, 616307, AR
ENSG00000035928	RFC1	protein_coding	102579	NA
ENSG00000206675	RNU6-32P	snRNA	NA	NA
ENSG00000222592	RNU6-887P	snRNA	NA	NA
ENSG00000134962	KLB	protein_coding	611135	NA
ENSG00000264621	MIR5591	miRNA	NA	NA
ENSG00000238797	Y_RNA	misc_RNA	NA	NA
ENSG00000163682	RPL9	protein_coding	603686	NA
ENSG00000121897	LIAS	protein_coding	607031	Hyperglycinemia, lactic acidosis, and seizures, 614462, AR
ENSG00000224097	RP11-472B18.1	pseudogene	NA	NA
ENSG00000109814	UGDH	protein_coding	603370	NA
ENSG00000249348	UGDH-AS1	antisense	NA	NA
ENSG00000163683	SMIM14	protein_coding	NA	NA
ENSG00000252796	RNU7-11P	snRNA	NA	NA
ENSG00000255458	RP11-539G18.2	lincRNA	NA	NA
ENSG00000078140	UBE2K	protein_coding	602846	NA
ENSG00000252975	Y_RNA	misc_RNA	NA	NA
ENSG00000249019	RP11-539G18.1	pseudogene	NA	NA
ENSG00000243260	RN7SL558P	misc_RNA	NA	NA
ENSG00000180610	ZBTB12P1	pseudogene	NA	NA

ENSG00000121892	PDS5A	protein_coding	613200	NA
ENSG00000271278	TCEB1P33	pseudogene	NA	NA
ENSG00000252970	RNA5SP159	rRNA	NA	NA
ENSG00000250568	RP11-333E13.2	pseudogene	NA	NA
ENSG00000231707	PABPC1P1	pseudogene	NA	NA
ENSG00000249064	KRT18P25	pseudogene	NA	NA
ENSG00000205794	RP11-333E13.4	pseudogene	NA	NA
ENSG00000078177	N4BP2	protein_coding	NA	NA
ENSG00000200455	RNU6-1112P	snRNA	NA	NA
ENSG00000201863	SNORA51	snoRNA	NA	NA
ENSG00000248977	RP11-395I6.1	pseudogene	NA	NA
ENSG00000260296	RP11-395I6.3	sense_overlapping	NA	NA
ENSG00000168421	RHOH	protein_coding	602037	{?Epidermodysplasia verruciformis, susceptibility to, 4}, 618307, AR
ENSG00000250338	RP11-395I6.2	lincRNA	NA	NA
ENSG00000249241	AC195454.1	lincRNA	NA	NA
ENSG00000174343	CHRNA9	protein_coding	605116	NA
ENSG00000239010	RNU7-74P	snRNA	NA	NA
ENSG00000250893	RP11-588L15.2	antisense	NA	NA

Table S2. Genes and known disease associations within the CANVAS linkage region

region	repeat.	pval	adj	Func.refGene	Gene.refGene
chr4:39350095-39350508	AAGGG	0.0027	1	intronic	RFC1
chr5:68499727-68500146	AATATATATATATAG	0.0027	1	intronic	CENPH
chr7:157344383-157344946	ACAGCCACCACCCACCCC	0.0027	1	intronic	PTPRN2
chr9:138739792-138740277	AAGGGGAGGGGAGTGGGGGG	0.0027	1	intronic	CAMSAP1
chr11:100495523-100496852	AATATGTGTATATATGT	0.0027	1	intergenic	CNTN5; LOC100128386
chr11:127149089-127150146	AAG	0.0027	1	ncRNA_intronic	LOC101929497
chr19:43901669-43901885	AAATATATATTATATAT	0.0027	1	intronic	TEX101
chr20:43066951-43067388	AAAAATATAATATAT	0.0027	1	intergenic	HNF4A; LINC01430
chr3:175416044-175416658	ACACATACATATAT	0.0028	1	intronic	NAALADL2
chr5:4286195-4286524	AAGCTATATATATATAGTG	0.0028	1	intergenic	IRX1; LINC02114
chr5:74613999-74614513	AAAG	0.0028	1	intergenic	ANKRD31; HMGCR
chr6:165352545-165353525	AAAG	0.0028	1	intergenic	MEAT6; C6orf118
chr7:4930864-4931703	AGAT	0.0028	1	intergenic	RADIL; MMD2
chr7:157846063-157847123	ACCCCAGAGACGCAGAG	0.0028	1	intronic	PTPRN2
chr10:134789654-134790481	AATACATTCCACGTGTATC	0.0028	1	ncRNA_exonic	LINC01168
chr11:2182294-2182840	ACACCCCTGTCCCC	0.0028	1	UTR5	INS; INS-IGF2
chr11:120746805-120747444	ACC	0.0028	1	ncRNA_intronic	LOC101929227
chr11:134177695-134179032	ACC	0.0028	1	intronic	GLB1L3
chr13:40788773-40788913	AATATAT	0.0028	1	ncRNA_intronic	LINC00548

Table S3: Expansion Hunter de novo results - screening for RE in two CANVAS individuals compared to 31 controls (all PCR based WGS, $p < 0.01$) ranked by p-value and ordered by chromosome

position	REF	ALT	genes	region	SNP	MAF	CANVAS1	CANVAS2	CANVAS3	CANVAS4	CANVAS6	CANVAS7	CANVAS8	CANVAS9	CANVAS10	CANVAS12	CANVAS18	CANVAS14	Core haplotype
38777173	A	T	TLR10	exonic	rs10856838	0.2806	0/0		0/0	0/1	0/1	1/1	0/0	0/0	0/0	0/1	0/1	0/0	
38777236	T	G	TLR10	UTR5	rs10856839	0.2803	0/0	0/0	0/0	0/1	0/1	1/1	0/0	0/0	0/0	0/1	0/1	0/0	
38798515	G	A	TLR1	exonic	rs72493538	0.0063	0/0	0/0	0/0	0/0	0/0	0/1	0/0	0/0	0/0	0/0	0/0	0/0	
38798648	C	A	TLR1	exonic	rs5743618	0.4732	0/0	0/1	0/0	1/1	0/0	1/1	0/0	1/1	0/0	1/1	1/1	1/1	
38798935	C	T	TLR1	exonic	rs5743614	0.3942	0/0	0/1	0/0	1/1	0/0	0/1	0/0	0/0	0/0	1/1	0/1	1/1	
38799269	G	A	TLR1	exonic	rs770320905	3.23E-05	0/0	0/0	0/0	0/1	0/0	0/0	0/0	0/0	0/0	0/0	0/0	0/0	
38799539	T	A	TLR1	exonic	rs3923647	0.0364	0/0	0/0	0/0	0/0	0/0	0/0	0/0	0/0	0/0	0/0	0/0	1/1	
38799710	T	C	TLR1	exonic	rs4833095	0.4041	0/0	0/1	0/0	1/1	0/0	0/1	0/0	0/0	0/0	1/1	0/1	1/1	
38800214	C	G	TLR1	exonic	rs5743611	0.1029	0/0	0/0	0/0	0/0	0/1	0/0	0/0	0/0	0/0	0/0	0/0	0/0	
38829163	A	C	TLR6	exonic	rs5743818	0.2286	1/1	0/0	0/1	0/0	0/1	0/0	0/0	0/0	0/0	0/0	0/0	0/0	
38829832	T	C	TLR6	exonic	rs3775073	0.4618	1/1	0/0	0/1	0/1	0/1	0/0	0/0	0/0	0/0	0/1	0/1	1/1	
38830012	G	C	TLR6	exonic	rs3821985	0.4546	1/1	0/0	0/1	0/1	0/1	0/0	0/0	0/0	0/0	0/1	0/1	1/1	
38830116	C	T	TLR6	exonic	rs3796508	0.0134	0/0	0/0	0/0	0/1	0/0	0/0	0/0	0/0	0/0	0/1	0/1	0/0	
38830350	A	G	TLR6	exonic	rs5743810	0.7116	1/1	0/1	1/1	1/1	0/1	1/1	0/0	0/0	0/0	1/1	1/1	1/1	
38830736	A	G	TLR6	exonic	rs5743808	0.0336	0/0	0/0	0/0	0/1	0/0	0/0	0/0	0/0	0/0	0/1	0/1	0/0	
38879655	C	T	FAM114A1	intronic	rs73236661	0.3071	1/1	0/0	0/1	0/1	0/1	0/0	0/0	0/0	0/0	0/0	0/1	0/0	
38879949	G	A	FAM114A1	exonic	rs11096964	0.308	1/1	0/0	0/1	0/1	0/1	0/0	0/0	0/0	0/0	0/0	0/1	0/0	
38880046	T	C	FAM114A1	exonic	rs11555334	0.3075	1/1	0/0	0/1	0/1	0/1	0/0	0/0	0/0	0/0	0/0	0/1	0/0	
38880136	A	T	FAM114A1	intronic	rs11943209	0.308	1/1	0/0	0/1	0/1	0/1	0/0	0/0	0/0	0/0	0/0	0/1	0/0	
38880167	C	T	FAM114A1	intronic	rs11938589	0.308	1/1	0/0	0/1	0/1	0/1	0/0	0/0	0/0	0/0	0/0	0/1	0/0	
38880181	A	ATGTGT	FAM114A1	intronic	rs111676993	0.3053	1/1	0/0	0/1	0/1	0/1	0/0	0/0	0/0	0/0	0/0	0/1	0/0	
38924358	A	G	FAM114A1	intronic	rs12498685	0.1954	1/1	0/0	0/1	0/0	0/0	1/1	0/0	0/0	0/0	0/0	0/0	0/0	
38930921	A	G	FAM114A1	exonic	rs3188469	0.1131	1/1	0/0	0/1	0/0	0/0	0/0	0/0	0/0	0/0	0/0	0/0	0/0	
38930985	T	TAA	FAM114A1	intronic	rs35369040	0.2194	0/0	0/0	0/1	0/1	0/0	0/0	0/0	1/1	0/0	0/0	0/0	0/0	
38930995	C	A	FAM114A1	intronic	rs58035134	0.2307	0/0	0/0	0/1	0/1	0/0	0/0	0/0	1/1	0/0	0/0	0/0	0/0	
38937372	T	C	FAM114A1	exonic	rs2271031	0.6742	1/1	0/0	1/1	1/1	1/1	1/1	0/1	1/1	0/0	0/0	0/0	0/0	
38944976	A	AT	FAM114A1	intronic	rs34903665	0.6885	1/1	0/1	0/1	0/1	1/1	0/0	0/1	1/1	0/0	0/1	0/1	0/0	
38945169	A	G	FAM114A1	exonic	rs1060582	0.1114	0/1	0/0	0/0	0/0	0/0	0/0	0/0	0/0	0/0	0/0	0/0	0/0	
38972691	T	C	TMEM156	exonic	rs140693293	0.0038	0/0	0/0	0/0	0/0	0/0	0/0	0/0	0/0	0/0	0/1	0/0	0/0	
38972793	A	G	TMEM156	intronic	rs3733268	0.0197	0/0	0/1	0/0	0/0	0/0	0/0	0/0	0/0	0/0	0/0	0/0	0/0	
38990334	C	T	TMEM156	intronic	rs3821986	0.4426	0/1	1/1	1/1	0/1	1/1	0/0	1/1	0/0	0/1	1/1	1/1	0/0	
38995310	A	G	TMEM156	intronic	rs11721954	0.3605	0/0	0/0	0/1	0/0	0/0	0/0	0/1	0/0	0/1	1/1	1/1	0/0	
38995374	T	C	TMEM156	exonic	rs10212770	0.88	0/1	1/1	1/1	1/1	1/1	1/1	1/1	1/1	1/1	1/1	1/1	0/0	**C**
39000305	A	G	TMEM156	exonic	rs11542133	0.23	0/0	0/0	0/0	0/0	0/0	0/0	0/0	0/0	0/1	0/1	0/0	0/0	**A**
39064126	G	C	KLHL5	UTR5	rs2711942	0.74	0/1	1/1	1/1	1/1	1/1	0/0	0/0	1/1	1/1	1/1	1/1	1/1	**C**
39064162	A	C	KLHL5	exonic	rs2711941	0.74	0/1	1/1	1/1	1/1	1/1	0/0	0/0	1/1	1/1	1/1	1/1	1/1	**C**
39077567	G	GT	KLHL5	intronic	rs201873328	0.02	0/0	0/0	0/0	0/0	0/0	0/0	0/0	0/0	0/1	0/0	0/0	0/0	**G**
39114929	G	A	KLHL5	intronic	rs3796510	0.53	0/0	0/0	0/0	0/0	0/0	0/0	0/1	0/0	0/0	0/0	0/0	1/1	**G**
39116775	A	T	KLHL5	intronic	rs10026775	0.53	0/0	0/0	0/0	0/0	0/0	0/0	0/1	0/0	0/0	0/0	0/0	1/1	**A**
39116911	T	C	KLHL5	exonic	rs3733276	0.53	0/0	0/0	0/0	0/0	0/0	0/0	0/1	0/0	0/0	0/0	0/0	1/1	**T**
39117103	G	A	KLHL5	intronic	rs3733277	0.53	0/0	0/0	0/0	0/0	0/0	0/0	0/1	0/0	0/0	0/0	0/0	1/1	**G**
39205365	C	T	WDR19	intronic	rs1451817	0.97	1/1	1/1	1/1	1/1	1/1	1/1	1/1	1/1	1/1	1/1	1/1	1/1	**T**
39216221	C	T	WDR19	exonic	rs2167494	0.29	0/1	0/0	0/0	0/0	0/0	0/0	0/1	0/0	0/0	0/0	0/0	0/0	**C**
39216240	G	A	WDR19	exonic	rs75964850	0.04	0/0	0/0	0/0	0/0	0/0	0/0	0/1	0/0	0/0	0/0	0/0	0/0	**G**
39217779	C	T	WDR19	exonic	rs199765304	0	0/0	0/0	0/0	0/0	0/0	0/0	0/1	0/0	0/0	0/0	0/0	0/0	**A**
39229771	G	A	WDR19	intronic	rs11730558	0.31	0/1	0/0	0/0	0/0	0/0	0/0	0/1	0/0	0/0	0/0	0/0	0/0	**G**
39242111	C	A	WDR19	intronic	rs9998591	0.64	0/1	1/1	1/1	1/1	1/1	1/1	0/1	1/1	1/1	1/1	1/1	1/1	**A**
39254828	A	C	WDR19	exonic	rs187546086	0	0/1	0/0	0/0	0/0	0/0	0/0	0/0	0/0	0/0	0/0	0/0	0/0	**A**
39255496	A	G	WDR19	intronic	rs33997556	0.08	0/1	1/1	1/1	1/1	1/1	1/1	0/1	1/1	1/1	1/1	1/1	0/0	**G**
39271541	A	G	WDR19	intronic	rs3733280	0.31	0/1	0/0	0/0	0/0	0/0	0/0	0/1	0/0	0/0	0/0	1/1	0/0	**A**
39276623	A	AG	WDR19	intronic	rs11096989	0.5	1/1	1/1	1/1	1/1	1/1	1/1	1/1	1/1	1/1	1/1	1/1	0/0	**AG**
39279724	T	C	WDR19	intronic	rs12648082	0.5	1/1	1/1	1/1	1/1	1/1	1/1	1/1	1/1	1/1	1/1	1/1	0/0	**C**
39279907	A	G	WDR19	intronic	rs2276888	0.46	0/0	0/0	0/0	0/0	0/0	0/0	0/0	0/0	0/0	0/0	0/0	1/1	**A**
39297211	C	T	RFC1	intronic	rs41547922	0	0/0	0/0	0/1	0/1	0/0	0/0	0/0	0/0	0/0	0/0	0/0	0/0	**C**
39301605	G	C	RFC1	intronic	rs2066788	0.51	1/1	1/1	1/1	1/1	1/1	1/1	1/1	1/1	1/1	1/1	1/1	0/0	**C**
39302029	T	C	RFC1	exonic	rs2066786	0.45	0/0	0/0	0/0	0/0	0/0	0/0	0/0	0/0	0/0	0/0	0/0	1/1	**T**
39303925	A	G	RFC1	exonic	rs2066782	0.1	1/1	1/1	1/1	1/1	1/1	1/1	0/1	1/1	1/1	1/1	0/0	0/0	**G**
39329102	G	A	RFC1	intronic	rs11096992	0.47	1/1	1/1	1/1	1/1	1/1	1/1	1/1	1/1	1/1	1/1	1/1	1/1	**A**
39353122	C	T	RFC1	intronic	rs4975007	0.98	1/1	1/1	1/1	1/1	1/1	1/1	1/1	1/1	1/1	1/1	1/1	1/1	**T**
39353137	C	T	RFC1	intronic	rs374311239	0	0/0	0/1	0/0	0/0	0/0	0/0	0/0	0/0	0/0	0/0	0/0	0/0	**C**
39439264	T	C	KLB	intronic	rs4975015	0.1707	0/1	0/0	0/1	0/0	0/0	0/0	1/1	1/1	1/1	0/0	0/0	1/1	**C**
39448529	G	A	KLB	exonic	rs17618244	0.1528	0/0	0/1	0/0	0/0	1/1	0/0	0/0	0/0	0/0	0/1	1/1	0/0	**G**
39448542	C	G	KLB	exonic	rs7685429	0.7326	1/1	1/1	0/1	1/1	1/1	1/1	0/0	1/1	1/1	0/1	1/1	0/0	**G**
39448586	C	T	KLB	exonic	rs35372803	0.0375	0/0	0/1	0/0	0/0	0/0	0/0	0/0	0/0	0/0	0/0	0/0	0/0	**C**
39450229	C	A	KLB	exonic	rs4975017	0.2881	0/1	0/0	0/0	0/0	0/0	0/0	0/0	0/0	1/1	0/0	0/0	0/0	
39450403	TGA	T	KLB	UTR3	rs112327399	0.0603	0/0	0/0	0/0	0/0	0/0	0/0	0/0	1/1	0/0	0/0	0/0	0/0	
39457857	T	C	RPL9	intronic	rs1015450	0.1489	0/1	0/1	0/0	0/1	0/0	0/0	0/0	0/0	0/0	0/0	0/0	0/0	
39457870	T	TG	RPL9	intronic	rs3216720	0.6465	1/1	1/1	0/1	1/1	1/1	1/1	0/0	0/0	1/1	0/1	1/1	0/0	
39457954	G	C	RPL9	intronic	rs117602443	6.00E-04	0/0	0/0	0/0	0/0	0/0	0/0	0/0	0/0	0/0	0/1	0/0	0/0	
39459724	T	C	RPL9	intronic	rs2687958	0.6479	1/1	1/1	0/1	1/1	1/1	1/1	0/0	0/0	1/1	0/1	1/1	0/0	
39460328	G	A	RPL9	intronic	rs73137448	0.0563	0/0	0/0	0/0	0/0	0/0	0/0	0/0	1/1	0/0	0/0	0/0	0/0	
39460352	C	G	RPL9	intronic	rs11550000	0.2497	0/1	0/1	0/0	0/1	0/0	0/0	0/0	1/1	0/0	0/0	0/0	0/0	
39460413	C	A	RPL9	intronic	rs2276889	0.0234	0/0	0/0	0/1	0/1	0/0	1/1	0/0	0/0	0/0	0/0	0/0	0/0	
39460490	C	G	RPL9	intronic	rs2276890	0.2844	0/1	0/0	0/0	0/0	0/0	0/0	0/0	0/0	1/1	0/0	0/0	0/0	
39460539	G	A	RPL9	UTR5	rs116433592	0.005	0/1	0/0	0/0	0/0	0/0	0/0	0/0	0/0	0/0	0/0	0/0	0/0	
39460572	G	A	LIAS\x3bRPL9	upstream	rs3821989	0.006	0/0	0/0	0/0	0/0	0/0	0/0	0/0	0/0	0/0	0/1	0/0	0/0	
39460797	G	A	LIAS	intronic	rs2687959	0.1826	0/0	0/1	0/1	0/1	1/1	1/1	0/0	0/0	0/0	0/1	1/1	0/0	
39466639	A	G	LIAS	intronic	rs76534325	0.0378	0/0	0/0	0/0	0/0	0/0	0/0	0/0	1/1	0/0	0/0	0/0	0/0	
39469279	A	T	LIAS	intronic	rs79572060	0.0233	0/0	0/0	0/1	0/1	0/0	1/1	0/0	0/0	0/0	0/0	0/0	0/0	
39472786	C	T	LIAS	intronic	rs79140300	0.0308	0/0	0/0	0/1	0/1	0/0	1/1	0/0	0/0	0/0	0/0	0/0	0/0	
39472796	T	A	LIAS	intronic	rs73137454	0.0747	0/0	0/0	0/0	0/0	0/0	0/0	0/0	1/1	0/0	0/0	0/0	0/0	
39472842	T	A	LIAS	intronic	rs2125314	0.3142	0/1	0/0	0/0	0/0	0/0	0/0	0/0	0/0	1/1	0/0	0/0	0/0	
39474868	G	A	LIAS	intronic	rs749915	0.236	0/1	0/1	0/0	0/1	0/0	0/0	0/0	1/1	0/0	0/0	0/0	0/0	
39482384	A	G	LOC401127	ncRNA exonic	rs10009841	0.0747	0/0	0/0	0/0	0/0	0/0	0/0	0/0	1/1	0/0	0/0	0/0	0/0	
39482440	G	A	LOC401127	ncRNA exonic	rs2276891	0.0232	0/0	0/0	0/1	0/1	0/0	1/1	0/0	0/0	0/0	0/0	0/0	0/0	
39482584	T	C	LOC401127	ncRNA exonic	rs17438520	0.0259	0/1	0/0	0/0	0/0	0/0	0/0	0/0	0/0	1/1	0/0	0/0	0/0	
39482627	T	C	LOC401127	ncRNA exonic	rs2276893	0.0232	0/0	0/0	0/1	0/1	0/0	1/1	0/0	0/0	0/0	0/0	0/0	0/0	
39483115	A	G	LOC401127	ncRNA exonic	rs2687980	0.7109	1/1	1/1	0/1	1/1	1/1	1/1	0/0	1/1	1/1	0/1	1/1	0/0	
39506144	C	T	UGDH	intronic	rs1379819	0.1465	0/0	0/0	0/0	0/0	0/0	0/0	0/0	0/0	0/0	1/1	0/0	0/0	
39506997	GA	G	UGDH	intronic	rs33942301	0.248	0/1	0/0	0/1	0/1	0/0	1/1	0/0	0/0	1/1	1/1	0/0	0/0	
39507083	G	T	UGDH	intronic	rs4975019	0.3746	0/1	0/0	0/1	1/1	0/0	1/1	0/0	0/0	1/1	1/1	0/0	0/0	
39510175	G	A	UGDH	intronic	rs6815780	0.2211	0/1	0/0	0/1	0/1	0/0	1/1	0/0	0/0	1/1	1/1	0/0	0/0	
39510264	A	G	UGDH	exonic	rs1129052	0.2203	0/1	0/0	0/1	0/1	0/0	1/1	0/0	0/0	1/1	1/1	0/0	0/0	
39511359	T	C	UGDH	intronic	rs6531722	0.2211	0/1	0/0	0/1	0/1	0/0	1/1	0/0	0/0	1/1	1/1	0/0	0/0	
39512347	T	C	UGDH	exonic	rs11544855	0.1145	0/0	0/0	0/1	0/0	0/0	0/0	1/1	0/0	0/0	0/0	0/0	1/1	
39515736	A	G	UGDH	exonic	rs10019532	0.213	0/1	0/0	0/1	0/1	0/0	1/1	0/0	0/0	1/1	1/1	0/0	0/0	
39515806	TAAAAAGA	T	UGDH	intronic	rs151116497	0.1451	0/0	0/0	0/0	0/0	0/0	0/0	0/0	0/0	0/0	1/1	0/0	0/0	
39528752	G	A	UGDH	intronic	rs4975020	0.7065	1/1	0/1	1/1	1/1	0/0	1/1	1/1	0/0	1/1	1/1	1/1	1/1	
39606900	T	C	SMIM14	intronic	rs147611359	0.0076	0/0	0/0	0/0	0/0	0/0	0/0	0/1	0/0	0/0	0/0	0/0	0/0	
39757480	A	AT	UBE2K	intronic	rs11389468	0.8191	1/1	1/1	0/1	1/1	0/0	1/1	0/1	1/1	0/1	1/1	1/1	1/1	
39757525	C	T	UBE2K	intronic	rs138858087	0.0072	0/0	0/0	0/0	0/0	0/0	0/0	0/1	0/0	0/0	0/0	0/0	0/0	
39871117	G	GA	PDS5A	intronic	rs34039605	0.3225	0/1	1/1	0/0	0/1	0/0	0/0	0/0	1/1	0/1	1/1	1/1	0/0	
39900041	T	C	PDS5A	exonic	rs193075238	3.24E-05	0/0	0/0	0/0	0/0	0/0	0/0	0/0	0/0	0/0	0/0	1/1	0/0	
39903924	G	A	PDS5A	intronic	rs77174235	0.0728	0/1	0/1	0/0	0/1	0/0	0/0	0/0	1/1	0/1	0/0	0/0	0/0	

Table S4. CANVAS core haplotype from WES data (core haplotype shown in dark green)
